# US FDA Advisory Panel Members’ Assessment of Premarket Approval Process and Suggestions for Improvement

**DOI:** 10.1001/jamanetworkopen.2024.36066

**Published:** 2024-10-09

**Authors:** Murad Alam, Victoria J. Shi, Amanda Maisel-Campbell, Brienne D. Cressey, Umer Nadir, Eric Koza, Misha Haq, Areeba Ahmed, Melissa S. Ma, Alexandra Weil, Brian A. Cahn, Angela Y. Lee, Sidney Shapiro, Emily Poon

**Affiliations:** 1 Department of Dermatology, Feinberg School of Medicine, Chicago, Illinois; 2 Department of Otolaryngology, Feinberg School of Medicine, Chicago, Illinois; 3 Department of Surgery, Feinberg School of Medicine, Chicago, Illinois; 4 Department of Medical Social Sciences, Feinberg School of Medicine, Chicago, Illinois; 5 Department of Dermatology, Columbia University Irving Medical Center, New York, New York; 6 Northeast Dermatology, Portsmouth, New Hampshire; 7 Department of Dermatology, University of Illinois, Chicago; 8 Marketing Department, Kellogg School of Management, Northwestern University, Chicago, Illinois; 9 Wake Forest University School of Law, Winston-Salem, North Carolina

## Abstract

**Importance:**

The manufacturing and marketing of medical devices is regulated by the US Food and Drug Administration (FDA), and the FDA premarket approval (PMA) process evaluates the safety and effectiveness of medical devices. The PMA process includes a detailed scientific, regulatory and quality system review and is critical to ensure that novel devices are safe, effective, and meet the needs of patients.

**Objective:**

To survey current voting members serving on panels of the FDA’s Medical Devices Advisory Committee to better characterize panel decision-making and identify steps for improvement.

**Design, Setting, and Participants:**

This qualitative survey study included 36 questions that were mailed to FDA device panelists regarding their opinions on the influence of sources of information, pivotal trial design, quality of evidence, panel composition and internal deliberative process, time allocation, and impartiality of the FDA. The survey was mailed to the members of all 18 FDA device panels in January and February 2017. Data were collected from January to May 2017 and analyzed from 2018 to 2019.

**Exposures:**

Respondents read and returned the aforementioned paper survey, while nonrespondents did not.

**Main Outcomes and Measures:**

The main outcomes included panel members’ perceptions, and their implications for process improvement. χ^2^ or Fisher exact tests were used to test differences between subgroups.

**Results:**

Of 64 of 92 panel members who responded (69.6%), 38 of 64 (59.4%) were male, 3 of 63 (4.8%) were Black respondents, 46 of 63 (73.0%) were White respondents, and 36 of 60 (60.0%) were in academic practice. The mean (range) panel service was 6.8 (1-22) years with 3.9 (1-19) meetings attended. Overall, respondents considered information presented by the FDA unbiased, and 28 of 61 (45.9%) believed that pivotal trials were frequently well-designed, 55 of 62 respondents (88.7%) suggested FDA consult panel members preemptively regarding trial design and 54 of 64 (84.4%) regarding the device label. Most indicated that prior FDA approval of another device serving the same medical purpose (43 of 62 [69.4%]) or approval in other countries with comparable regulatory regimes, such as Canada and Europe (39 of 62 [62.9%]), would make them more likely to recommend approval. Respondents rated written information (50 of 60 [83.3%]), live presentations (43 of 58 [74.1%]), and prior professional knowledge (41 of 60 [68.3%]) as the most important sources of information in deciding whether to recommend approval. Additionally, 52 of 58 respondents (89.7%) recommended that a panel member–only executive session would allow more clarity and honesty in deliberations, and 33 of 59 (55.9%) believed a three-fourths majority appropriate for recommending approval, which would be a deviation from the current system in which an overall vote is reported without designation of a vote threshold.

**Conclusions and Relevance:**

In this survey study of FDA device panel members, respondents wanted improved study designs, more relevant clinical data, including from other countries, involvement of panelists in study design and device label development, and inclusion of an executive session. Demographically, panels could be made more diverse.

## Key Points

**Question:**

What are the perceptions of voting members serving on US Food and Drug Administration (FDA) device panels regarding the FDA’s medical device premarket approval process and potential improvements?

**Findings:**

In this survey study of 64 device panel members, 46% noted that pivotal trials were frequently well-designed, and 89% and 84% suggested the FDA preemptively consult panel members about trial design and device labels, respectively. To allow more clarity and honesty in deliberations, 87% of the panelists recommended having an executive session that included only panel members.

**Meaning:**

These findings suggest that the FDA premarket approval process may be improved by increasing the role of panelists.

## Teaser

This survey study examines the opinions of US Food and Drug Administration (FDA) panel members regarding the premarket approval process for devices.

## Introduction

The US Food and Drug Administration (FDA) regulates the manufacturing and marketing of medical devices through the Center for Devices and Radiological Health.^1,2^ The goal of the FDA premarket approval (PMA) process is to evaluate the safety and effectiveness of medical devices.^3^ The PMA is an in-depth process that includes a detailed scientific, regulatory, and quality system review. PMAs are required for novel class III medical devices (ie, those that support or sustain human life) and are of substantial importance to prevent impairment of human health or identify those that may present a potential, unreasonable risk of illness or injury. A PMA is not necessary when a class III device is substantially equivalent to an already legally marketed device (ie, a predicate device, defined in section 513[i][1][A] Food Drug and Cosmetic Act). In this case, a premarket notification, or 510(k), is submitted to the FDA, and the submitter receives a letter from the FDA, which confirms substantial equivalence and clears the device for marketing in the US. Finally, most class I and class II devices are exempt from premarket notification requirements. While only a small minority of novel class III devices undergo the PMA process, the importance of ensuring that novel devices are safe and effective means that the PMA process is critical to ensure that medical devices in the US best meet the needs of patients.

Prior to approval of class III novel devices without a predicate device, FDA staff may convene a panel of independent experts to recommend whether approval is warranted. Because the FDA’s Medical Devices Advisory Committee (MDAC) panel reviews are time and labor intensive, not all novel devices may be afforded a panel review. The MDAC consists of 18 panels, each with 6 to 7 voting members selected based on expertise and lack of conflicts for fixed terms, as well as additional temporary voting members and nonvoting members. Panels review pivotal trials (ie, the primary trials used to make approval decisions), other industry data, and FDA assessments to advise whether the device under review meets the 21CFR860.7 criteria of reasonable assurance of safety and effectiveness.

While PMAs are only required for novel devices, the PMA process is crucial in ensuring that such devices are appropriately approved. Since the FDA usually accepts panel recommendations and these may be foundation for subsequent 510(k) submissions of similar devices, it is imperative that panels have all the information they need to make a recommendation and that their internal process is rigorous. Indeed, recently the FDA commissioner noted the importance of revising the advisory committee meeting structure to ensure more robust discussion and to include evidence from clinical practice in deliberations, among other potential reforms.^4^ This study surveyed current voting members serving on panels of the MDAC to better characterize panel decision-making processes and identify steps for improvement.

## Methods

### Study Design

This study was a paper-based survey of US FDA MDAC panelists. The study protocol was reviewed by the institutional review board at Northwestern University and deemed exempt. Because this survey was deemed exempt, informed consent was not required from the study participants. Reporting of this study was in accordance with the American Association for Public Opinion Research (AAPOR) reporting guideline^5^ for survey studies.

### Survey Development

The survey was developed based on a qualitative review of the FDA PMA process, and review of key elements of the process, as obtained from transcripts of panel meetings stored on the FDA site. Individual items on the survey were refined in consultation with the University of Chicago Survey Lab, which provided expertise on restating items to improve clarity, optimize the quality of data collected, and minimize bias. The survey instrument included 36 questions regarding the relative influence of sources of information, pivotal trial design, quality of evidence, panel composition and internal deliberative process, time allocation, and impartiality of the FDA (eMethods in Supplement 1). Members’ demographic features and duration of service were recorded. Race and ethnicity information was self-reported. The options regarding race and ethnicity were American Indian or Alaska Native, Asian, Black or African American, Native Hawaiian or other Pacific Islander, and White or Caucasian. Race and ethnicity were assessed to better understand whether panelists were representative of the general population and the degree to which there was diversity within panels. Items were developed in collaboration with academic experts in transparency and group dynamics (A.L.), a technical advisor on survey design (M.V.), a current voting member of the MDAC (M.A.), and other members of the MDAC who reviewed the survey for content.

### Survey Participants

Voting members of any MDAC panel, per the FDA website, were eligible, provided that they had attended at least 1 panel meeting, per the meeting minutes. Designated federal officers and industry and consumer representatives were excluded as they were not voting. Additionally, 1 member leading survey development (M.A.) was excluded.

### Survey Administration and Data Collection

The cover letter, survey, and prepaid return envelope were sent by overnight mail in January to February 2017 to panelists, using addresses listed under on the FDA website. Individuals who did not respond within 2 weeks (ie, nonresponders) received an email to confirm their mailing address and were sent a duplicate survey. Follow-up emails were sent at 4 weeks and 6 weeks. No financial incentive was provided.

### Statistical Analysis

Completed surveys were defined as having at least 50% of nondemographic items answered and were analyzed using SAS version 3.71 (SAS Institute). Descriptive statistics were performed, and respondent demographics were compared with nonresponders using 2-tailed χ^2^. Data were collected from January to May 2017.

We compared differences in decision-making based on sex and level of experience (ie, 1 to 3 meetings attended vs more). While there is variation in how often particular panels are convened, FDA device panels typically meet several times a year, so panelists attending fewer meetings than expected in a year were deemed less experienced. We tested the impact of information sources available for panel decisions on these outcomes. Likert scale responses were dichotomized for analysis given the small sample size: (1) not at all, somewhat, or moderately influential vs very or extremely influential; (2) not important or a little important vs moderately or very important; or (3) less likely, just as likely, or a little more likely vs moderately or much more likely. χ^2^ or Fisher exact tests were used to test differences between subgroups. *P* < .05 were considered significant. Data were analyzed from 2018 to 2019.

## Results

Names and mailing addresses for 99 panelists were obtained, 7 were excluded for not having attended a panel meeting, and 71 of 92 panelists (77.1%) returned surveys, of which 7 were less than 50% completed and excluded. This led to an adjusted response rate of 64 of 92 (69.6%). Of the respondents, 38 of 64 (59.4%) were male, 3 of 63 (4.8%) were Black respondents, 46 of 63 (73.0%) were White respondents, and 36 of 60 (60.0%) were in academic practice. Analysis revealed no significant differences between respondents and the total sample in sex, region, or specific MDAC panel. The mean (range) panel service was 6.8 (1-22) years with 3.9 (1-19) meetings attended (eTable 1 in Supplement 1).

### Relative Influence of Information Available for Panel Decisions

Panelists agreed that written information (50 of 60 [83.3%]), live presentations (43 of 58 [74.1%]), and prior professional knowledge of the device-under review (41 of 60 [68.3%]) were more important sources of information regarding device approval, in contrast to public comments (10 of 60 [16.6%]) (Table 1 and eTable 2 in Supplement 1). Comments from organization or group spokespersons were significantly more important than those from individuals (78.2% vs 21.8%; *P* < .001). More experienced members (who had attended more than 3 meetings) were more likely to rate live presentations (91.3% vs 59.4%; *P* = .01) and industry responses to panel questions (62.5% vs 33.3%; *P* = .03) as very or extremely important compared with those with less experience (eTable 3 in Supplement 1).

**Table 1.** Relative Influence of Information Available for Panel Decisions

Relative influence of information available for panel decisions	Participant, No. (%)				
	Not at all influential	Somewhat influential	Moderately influential	Very influential	Extremely influential
Written information read prior to the meeting (n = 60)	0	2 (3.3)	8 (13.3)	22 (36.7)	28 (46.7)
Live presentations to the panel (n = 58)	0	2 (3.5)	13 (22.4)	25 (43.1)	18 (31.0)
Prior professional knowledge (n = 60)	0	3 (5.0)	16 (26.7)	24 (40.0)	17 (28.3)
Other panel member opinions expressed during the panel review (n = 60)	0	5 (8.3)	10 (16.7)	31 (51.7)	14 (23.3)
Outside responses (FDA) to panel questions during the presentation (n = 60)	1 (1.7)	6 (10.0)	16 (26.7)	23 (38.3)	14 (23.3)
Comparison data for existing approved devices (n = 58)	1 (1.6)	3 (5.2)	19 (32.8)	23 (39.7)	12 (20.7)
Outside responses (industry) to the panel questions during the presentation (n = 60)	2 (3.4)	5 (8.3)	24 (40.0)	17 (28.3)	12 (20.0)
Public comments made by citizens, patients and professional societies and others during the comment period (n = 60)	7 (11.7)	21 (35.0)	22 (36.7)	8 (13.3)	2 (3.3)

Abbreviation: FDA, US Food and Drug Administration.

Respondents reported that written information from the FDA was more important than sponsor or company information (79.3% vs 22.4%; *P* < .001) and considered live FDA presentations more important than sponsor presentations (64.4% vs 35.6%; *P* = .02). No difference in importance was reported between FDA and sponsor responses to panel member questions (eTable 4 in Supplement 1). Although most respondents weighed safety and effectiveness equally in recommendations for approval (34 of 59 [57.6%]), 19 of 59 respondents (32.2%) considered safety more important and 6 of 59 (10.2%) considered effectiveness more important (eTable 5 in Supplement 1).

Of respondents, 39 of 62 (62.9%) would be more likely to recommend approval of a device already approved in another industrialized country (ie, Canada, Europe) (eTable 6 in Supplement 1). Furthermore, 31 of 62 (50.0%) would be more likely to recommend approval of a device already approved for another indication. Most respondents (43 of 62 [69.4%]) indicated that prior FDA approval of another device serving the same medical purpose with comparable effectiveness and safety would make them more likely to recommend approval.

### Pivotal Trial Research Designs

Additionally, 28 of 61 respondents, (45.9%) indicated that pivotal trials for devices were frequently well-designed, while 55 of 62 (88.7%) believed it would be helpful for the FDA to solicit panel members’ advice regarding the appropriate design of pivotal trials. Most (54 of 64 [84.4%]) also recommended that the FDA should have panel members review the device label (eTables 7 to 9 in Supplement 1).

### Depth and Detail of Evidence Provided

Among respondents, 43 of 57 (75.4%) indicated that the totality of the scientific evidence presented to the panel was often or almost always sufficient to make them feel very comfortable making a decision recommending device approval or nonapproval (Table 2 and eTable 10 in Supplement 1). However, many considered too superficial information pertaining to serious adverse events (21 of 61 [34.4%]), patients who did unusually poorly (21 of 61 [34.4%]), and long-term use considerations or outcomes (25 of 59 [42.4%]).

**Table 2.** Respondents’ Beliefs About the Adequacy of the Typical Level of Detail Provided for Evidence Relevant to Their Decision to Recommend Approval or Disapproval of a Medical Device

Type of evidence	Respondents, No. (%)					
	Much too superficial	A little too superficial	About right	A little too detailed	Much too detailed	Too varied to say
In vitro or animal studies (n = 59)	3 (5.1)	5 (8.5)	36 (61.0)	7 (11.9)	0	8 (13.6)
Epidemiologic and clinical information about condition device treats (n = 60)	0	11 (18.3)	38 (63.3)	4 (6.7)	0	7 (11.7)
Safety data vis-à-vis approved devices for condition (n = 61)	0	11 (18.0)	42 (68.9)	2 (3.3)	1 (1.6)	5 (8.2)
Patients who did unusually well (n = 61)	1 (1.7)	11 (18.0)	33 (54.1)	6 (9.8)	3 (4.9)	7 (11.5)
Effectiveness data vis-à-vis approved devices for condition (n = 61)	1 (1.7)	11 (18.0)	39 (63.9)	4 (6.6)	1 (1.6)	5 (8.2)
Research design or biostatistical analysis of pivotal trial (n = 61)	0	15 (24.6)	36 (59.0)	5 (8.2)	1 (1.6)	4 (6.6)
Serious adverse events or patients who did unusually poorly (n = 61)	1 (1.7)	20 (32.8)	29 (47.5)	4 (6.6)	1 (1.6)	6 (9.8)
Long-term use considerations or outcomes (n = 59)	4 (6.7)	21 (35.6)	23 (39.0)	3 (5.1)	0	8 (13.6)

### Time Allocation

While 44 of 58 (75.9%) considered the time allotted to different panel meeting segments and the total duration to be about right (eTable 11 in Supplement 1), 19 of 58 (32.8%) found the duration of public commentary excessive. Conversely, 14 of 58 (24.1%) considered the time for panel members to discuss among themselves insufficient.

### Panel Process

Additionally, 52 of 58 respondents (89.7%) believed an executive session that included only panel members could potentially facilitate discussion prior to voting. Respondents indicated that such a session would allow for more clarity (43 of 57 [75.4%]) and honesty (41 of 57 [71.9%]) from panel members. On the other hand, 15 of 57 (26.3%) expressed concern that an executive session could prolong an already lengthy process (Table 3).

**Table 3.** Respondents’ Beliefs About the Helpfulness or Unhelpfulness of Having an Executive Session Prior to Voting (n = 58)^a^

Responses	Respondents, No. (%)
**Reasons why an executive session would be helpful (n=57)^b^**	
Allow for more clarity of panel opinions	43 (75.4)
Allow for more honesty of panel opinions	41 (71.9)
Inform better panelist question-asking	32 (56.1)
Help you decide how best to vote	21 (36.8)
Helpful- other	4 (7.0)
**Reasons why an executive session would be unhelpful (n=57)^b^**	
Prolong an already length process	15 (26.3)
Add pressure to agree with majority	8 (14.0)
Open opportunities to get lost in detail	8 (14.0)
Further cloud your voting decision	2 (3.5)
Unhelpful, other	2 (3.5)

^a^ Executive sessions were ones in which the room was cleared except for panel members to provide time for panel-only discussion. Respondent survey selection regarding the helpfulness or unhelpfulness of executive sessions: very helpful, 25 (43.1%); moderately helpful, 13 (22.4%); a little helpful, 14 (24.1%); have no effect, 4 (6.9%); a little unhelpful, 2 (3.4%); moderately unhelpful, 0; very unhelpful, 0; other, 2 (3.4). Total is more than 100% because at least 1 respondent selected more than 1 answer choice.

^b^ Respondents were asked to select all that apply.

Furthermore, 33 of 59 (55.9%) believed that a three-fourths majority among panel members would be preferable for recommending approval. Of note, while only 2 of 59 (3.4%) considered a unanimous vote appropriate, 13 of 59 (21.8%) perceived that the FDA would like a unanimous vote, while 1 of 59 (1.7%) perceived the sponsor or company to want unanimity (Figure; eFigure 1, eTable 12, and eTable 13 in Supplement 1). In contrast, 39 of 59 (66.1%) believed the sponsor or company would consider a simple majority sufficient to recommend approval.

**Figure.**
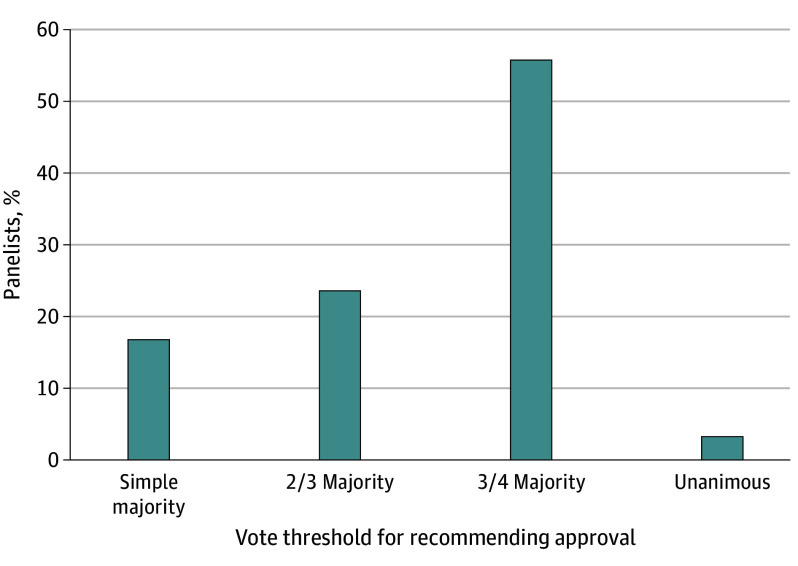
Level of Consensus Preferred by Panelists Respondents’ beliefs about the percentage of approval votes among panel members that they think would be appropriate for the panel to recommend approval.

## Discussion

While previous studies have examined the attitudes of physicians toward the US FDA’s medical device premarket approval process, to our knowledge, this is the first study of panel members’ perceptions of the FDA medical device premarket approval process and how this process could be improved. Among the major concerns raised by panelists were that pivotal studies were frequently not well-designed, and that device labels, which were developed by industry sponsors, were also suboptimal. A large majority suggested that the FDA should solicit the advice of panel members regarding the design of pivotal trials before these were authorized and conducted. Most panelists also suggested that the FDA ask the panel to review the device label proposed by a sponsor or company, and to offer recommendations regarding the information that should be included in the label. Importantly, most panelists also believed that it would be useful to add an executive session to panel meetings, which would be a period before voting during panel meetings when only the panelists were present. Executive sessions could enable more frank and honest discussion.

Most respondents did not believe that pivotal trials were frequently well-designed, and many expressed a desire for more adequately powered studies. This was consistent with concerns about the statistical analysis of device pivotal trials raised by others.^6^ The recommended time for improvement was before trials were authorized by the FDA and conducted by the sponsor. Having information from trials in other countries may have helped panelists avoid the necessity of basing recommendations solely on substandard US studies. However, such foreign studies may not have met other benchmarks for quality and would have been conducted on populations outside the US.

Comments by respondents indicated a frequent desire for more information about study design, power, and data analyses, including assessment by an independent statistician. Respondents also wanted more information regarding clinical experience during clinical trials with the device, including its use for the entire spectrum of the condition.

Panelists’ dismissal of the importance of patient and public perspectives was striking. While this needs to be explored further, it may not represent disparagement of the patient perspective but rather an acknowledgment that patient groups and others requesting time to speak at a panel meeting often have specific interests, preferences, and agendas that may not help panelists with their task of trying to assess the safety and effectiveness of a novel device. Those who wanted fewer public comments deemed these speeches to be carefully placed speakers from the sponsor and patient anecdotes intended to sway the panel (Table 4 and eTable 14 in Supplement 1).

**Table 4.** Respondents’ Comments Regarding Information They Routinely Wished for More or Less of in Order to Make a Good Decision

Theme (No.)	Details
**Respondents wanted more of these types of information (n=23)**	
Pivotal trials and data (9)	Details of study design, power analysis, and data analysis; analysis of study design, power, etc, from an expert statistician; patient demographics pertaining to device use and trials; adequately powered and designed studies; histopathology reports from preclinical trials; details regarding significant adverse events
Other sources of information (7)	How the device works; disclosure of sources of funding for groups presenting public comments; clinical experience regarding use of the device; opinions from patients; key published information from peer-reviewed journals; comparisons to other treatment options for condition; application of the device to the entire spectrum of condition
Postmarket surveillance or long-term effects (4)	Options for postmarket surveillance of approved devices to enhance safety; long-term outcomes (especially for permanent devices)
Panel meeting process or preparation (3)	Time at the panel meeting; advance notice about meetings and more information prior to meeting; preparation by the FDA about the process and role of panel members
**Respondents wanted less of these types of information (n = 9)**	
Public comments or patient anecdotes (5)	“Public comments by carefully placed speakers from sponsor- feels like commercial advertising”; “less individual patients. They provide anecdotal evidence intended to sway the panel.”
Specific sources of data or scientific background (4)	“Overly detailed stats background by the FDA”; “detailed info from the FDA-compiled by individuals with no clinical knowledge”; “irrelevant animal studies”; “data on very old predicate device-often useless and irrelevant”

Abbreviation: FDA, Food and Drug Administration.

Concerns regarding panel meeting transparency, which have been raised by other investigators,^7^ were apparent in responses. While some transparency is needed to minimize bias, prevent corruption, and restrict external influences on panel members, excessive transparency may compromise the quality of panel deliberations and hinder frank discussion.^8^ Grandwohl et al^8^ showed that regardless of the mechanisms used by a committee to aggregate information, requiring a high level of transparency during committee deliberations such that a decision-maker observes all actions taken by the committee, typically makes it “impossible for the committee to convey any [useful] information to the DM [Decision Maker].” Notably, the FDA’s ability to implement an executive session would be limited by the Federal Advisory Committee Act (FACA), which would likely not allow closure of panel meetings (eFigure 2 in Supplement 1). Moreover, it is improbable that the benefits of such an executive session alone would warrant the extraordinary step of asking Congress to step in and amend FACA. However, if a multistakeholder process involving patients, sponsors, the FDA, panelists, and the public were undertaken and eventually led to consensus regarding a series of reforms, including a panel executive session, legislative action to implement these reforms collectively may potentially be feasible.

While there is currently no requirement for a vote from an advisory committee for device approval and no set threshold for such a vote if it is taken, most respondents noted that they would feel most comfortable if a supermajority of panelists were in favor of approval. This suggests that panel members may consider even a few dissenting votes concerning and indicative of doubts regarding device approval. Notably, while the FDA does not have the regulatory authority to approve with reservations, panels’ recommendations to the FDA could potentially be more nuanced and include such an option. It may also be the case that the interests of the FDA and panelists may diverge in this respect, with panelists being primarily focused on scientific rigor and evaluating the evidence and the FDA having to consider the opinions of patient groups and sponsors eager for new device approvals, the clarity and consistency of messaging, and the political milieu of device approvals.

Recent research on FDA advisory committees shows that while panel recommendations are usually accepted by the FDA, the FDA has wide latitude regarding whether to convene panel meetings at all.^9,10^ Sometimes the logic is relatively clear, such as when FDA Summaries frequently state that, “In accordance with the provisions of section 515(c)(2) of the act as amended by the Safe Medical Devices Act of 1990, this PMA was not referred to the…FDA advisory committee, for review and recommendations because the information in the PMA substantially duplicates information previously reviewed by this panel.” There may be other reasons for avoiding panel meetings, as these are resource intensive, may create other burdens for FDA staff and leadership, and are increasingly convened less often. Just as other commentators have asked the FDA to be more consistent and transparent regarding the process of whether to convene panels, respondents in the current survey appeared to urge the FDA to make better use of their expertise when a panel was convened by involving them in pivotal trial designs, allowing them to have input into device labels, and sharing data from trials in other countries with comparable regulatory regimes with them.^11^ While respondents had a high degree of respect for the FDA and the integrity of the panel process, they seemed eager to be more helpful to the FDA. Notably, this may derive from altruistic self-selection, since panelists, who are carefully vetted for conflicts of interest and relationships with industry and are paid nominal to negligible amounts for their participation, may be highly motivated by a desire to protect and support patients and public health.

### Limitations

This study has limitations. While not all panel members responded, the demographic similarity and level of experience of respondents to nonrespondents indicate that responses were likely representative. Although how panel members are selected and whether panels are comprised of ideal members from an objective or professionally diverse perspective was outside our scope, the data also showed that panels could be made more diverse, as voting members on device panels appear to be mainly male, White individuals, and in academic practice. Social desirability bias may have influenced the responses of particular survey respondents. The wording of the questionnaire instrument was occasionally imperfect, as questions of regarding whether respondents would be prone to approve devices under certain circumstances should have been better stated as whether respondents would recommend these approvals, as panels can only recommend and the FDA decides. Also, the panelists’ opinions regarding postapproval or postmarketing (ie, phase 4) studies were not elicited because this is a rapidly evolving area with little enforcement and these studies do not typically impact the decision of whether or not to recommend approval.

## Conclusions

This survey study assessed the opinions of MDAC panel members regarding the FDA panel process. Panelists were eager to be more engaged in the approval process by potentially vetting pivotal study designs before clinical trial inception; reviewing more detailed information about adverse events, long-term outcomes, and trials conducted in other countries with similarly rigorous regulatory regimes; and having input on sponsor-generated device labels. It is unclear if the FDA has the resources or willingness to involve panelists in these tasks, but further consideration may be warranted. Panelists also wanted the FDA to consider changes that may be impractical in the current governing legislative framework. Specifically, FACA may preclude panelists from having a closed executive session in which panelists could speak to each other, and the FDA is not bound to consider any voting threshold of a panel meeting to be definitive, let alone the supermajority recommended by the respondents. A final thought may be that the PMA process may benefit from a collaborative, consensus process that brings together key stakeholders, including patients, support groups, scientists, FDA, clinicians, industry representatives, panelists, public members, and others to suggest improvements to the panel process and FACA. Like panelists, many of these groups are highly invested in device approvals and there may be important, creative solutions that make the process better for all.

## Notes

Supplement 1. **eMethods.** FDA CDRH Advisory Panel Survey **eTable 1.** Characteristics of Respondents and Nonrespondents **eTable 2.** Responses Regarding the Importance of Sponsor or Company Presentations **eTable 3.** Respondents in Each Gender and Experience Subgroup Who Selected “Very influential” or “Extremely Influential” for the Importance of Each Source of Information in Recommending Approval or Disapproval of New Devices **eTable 4**. Most Important Sources of Information Available for Panel Decisions **eTable 5.** Relative Weight of Safety and Effectiveness in Determining Suitability for Approval **eTable 6.** Responses to Questions Regarding Suitability for Approval Given the Following Hypothetical Scenarios Related to Device Safety and Efficacy **eTable 7.** Respondents’ Beliefs About Likelihood that Pivotal Trials Were Well-Designed with Regard to All Devices They Had Reviewed and Voted on at Panel Meetings **eTable 8.** Respondents’ Attitudes Toward the Helpfulness of Including Panel Members in Specific Approval Preparation Steps **eTable 9.** Responses to Questions Regarding Suitability for Approval Given the Following Hypothetical Scenarios Related to Pivotal Trials **eTable 10. **Respondents’ Beliefs About How Often the Scientific Evidence Makes Them Feel Comfortable About Deciding Device Approval or Nonapproval **eTable 11.** Respondents’ Beliefs about the Adequacy of the Time Allotted to Specific Segments of the Panel Meetings **eFigure 1.** Respondents’ Beliefs About the Percentage of Approval Votes Among Panel Members that the FDA and the Sponsor/Company Think Would Be Appropriate for the Panel to Recommend Approval **eTable 12.** Respondents’ Beliefs about the Adequacy of the Number of Participants Comprising the Average Voting Panel **eTable 13.** Responses to Questions Regarding Suitability for Approval Given the Following Hypothetical Scenario Related to Panel Voting **eTable 14.** Respondents’ Beliefs About the Extent to Which FDA Considers Harms versus Benefits, and is Inclined to Favor Device Approval **eFigure 2.** Impact of FACA on Potential Executive Sessions

Supplement 2.
Data Sharing Statement
