# Supplementary material for: US FDA Advisory Panel Members’ Assessment of Premarket Approval Process and Suggestions for Improvement
Source: JAMA Netw Open. 2024 Oct 9;7(10):e2436066. doi: 10.1001/jamanetworkopen.2024.36066 (PMC11581643; doi:10.1001/jamanetworkopen.2024.36066)
Supplement: Supplement 1. — eMethods. FDA CDRH Advisory Panel Survey eTable 1. Characteristics of Respondents and Nonrespondents eTable 2. Responses Regarding the Importance of Sponsor or Company Presentations eTable 3. Respondents in Each Gender and Experience Subgroup Who Selected “Very influential” or “Extremely Influential” for the Importance of Each Source of Information in Recommending Approval or Disapproval of New Devices eTable 4. Most Important Sources of Information Available for Panel Decisions eTable 5. Relative Weight of Safety and Effectiveness in Determining Suitability for Approval eTable 6. Responses to Questions Regarding Suitability for Approval Given the Following Hypothetical Scenarios Related to Device Safety and Efficacy eTable 7. Respondents’ Beliefs About Likelihood that Pivotal Trials Were Well-Designed with Regard to All Devices They Had Reviewed and Voted on at Panel Meetings eTable 8. Respondents’ Attitudes Toward the Helpfulness of Including Panel Members in Specific Approval Preparation Steps eTable 9. Responses to Questions Regarding Suitability for Approval Given the Following Hypothetical Scenarios Related to Pivotal Trials eTable 10. Respondents’ Beliefs About How Often the Scientific Evidence Makes Them Feel Comfortable About Deciding Device Approval or Nonapproval eTable 11. Respondents’ Beliefs about the Adequacy of the Time Allotted to Specific Segments of the Panel Meetings eFigure 1. Respondents’ Beliefs About the Percentage of Approval Votes Among Panel Members that the FDA and the Sponsor/Company Think Would Be Appropriate for the Panel to Recommend Approval eTable 12. Respondents’ Beliefs about the Adequacy of the Number of Participants Comprising the Average Voting Panel eTable 13. Responses to Questions Regarding Suitability for Approval Given the Following Hypothetical Scenario Related to Panel Voting eTable 14. Respondents’ Beliefs About the Extent to Which FDA Considers Harms versus Benefits, and is Inclined to Favor Device Approval eFigure 2. [file jamanetwopen-e2436066-s001.pdf]

# Supplemental Online Content

Alam M, Shi VJ, Maisel-Campbell A, et al. US FDA advisory panel members' assessment of premarket approval process and suggestions for improvement. *JAMA Netw Open*. 2024;7(9):e2436066.
doi:10.1001/jamanetworkopen.2024.36066

**eMethods.** FDA CDRH Advisory Panel Survey

**eTable 1.** Characteristics of Respondents and Nonrespondents

**eTable 2.** Responses Regarding the Importance of Sponsor or Company Presentations

**eTable 3.** Respondents in Each Gender and Experience Subgroup Who Selected "Very influential" or "Extremely Influential" for the Importance of Each Source of Information in Recommending Approval or Disapproval of New Devices

**eTable 4.** Most Important Sources of Information Available for Panel Decisions

**eTable 5.** Relative Weight of Safety and Effectiveness in Determining Suitability for Approval

**eTable 6.** Responses to Questions Regarding Suitability for Approval Given the Following Hypothetical Scenarios Related to Device Safety and Efficacy

**eTable 7.** Respondents' Beliefs About Likelihood that Pivotal Trials Were Well-Designed with Regard to All Devices They Had Reviewed and Voted on at Panel Meetings

**eTable 8.** Respondents' Attitudes Toward the Helpfulness of Including Panel Members in Specific Approval Preparation Steps

**eTable 9.** Responses to Questions Regarding Suitability for Approval Given the Following Hypothetical Scenarios Related to Pivotal Trials

**eTable 10.** Respondents' Beliefs About How Often the Scientific Evidence Makes Them Feel Comfortable About Deciding Device Approval or Nonapproval

**eTable 11.** Respondents' Beliefs about the Adequacy of the Time Allotted to Specific Segments of the Panel Meetings

**eFigure 1.** Respondents' Beliefs About the Percentage of Approval Votes Among Panel Members that the FDA and the Sponsor/Company Think Would Be Appropriate for the Panel to Recommend Approval

**eTable 12.** Respondents' Beliefs about the Adequacy of the Number of Participants Comprising the Average Voting Panel

**eTable 13.** Responses to Questions Regarding Suitability for Approval Given the Followin Hypothetical Scenario Related to Panel Voting

**eTable 14.** Respondents' Beliefs About the Extent to Which FDA Considers Harms versus Benefits, and is Inclined to Favor Device Approval

**eFigure 2.** Impact of FACA on Potential Executive Sessions

This supplemental material has been provided by the authors to give readers additional information about their work.

© 2024 Alam M et al. *JAMA Network Open.*

# FDA CDRH Advisory Panel Survey

**This 30 minute survey aims to help the FDA leadership better understand how well the current process for medical device advisory panels is working.**

This is a short survey that is part of a research study Questionnaire for FDA Advisory Board Panel Members.

Please note that:

- There are ***no*** panel or device-specific questions – this is about the ***overall process***.
- Completion of this survey does ***not*** affect your service or eligibility on CDRH panels in any way.
- This survey was ***independently developed*** by a group of panel members. The survey is ***not*** directly or indirectly affiliated or influenced by any corporate entity or other group with business before CDRH.
- ***FDA is aware*** that this survey is being conducted, and is interested in receiving the summary results, but FDA has had no input into the development of the survey and has neither approved or disapproved any questions on the survey.

Please also note:

- This survey is ***confidential***. All responses are separated from contact information and only unidentified data are retained.
- Your participation is critical but completely ***voluntary***. You may skip any questions you do not wish to answer and may end your participation at any time.
- This survey has been approved by the **Northwestern University Institutional Review Board (IRB)** (STU00204096), a set of committees that protect the rights of people who take part in research.
- ***Summary survey results will be emailed to all Panel members and forwarded to FDA leadership.***

**After you have completed this survey, please return it in the enclosed prepaid, preaddressed FedEx envelope.**

Should you have any questions, concerns or complaints regarding this research, you may contact Dr. Murad Alam, who is in charge of this research, at (312) 695-6829.

Should you have any questions about your rights as a subject in this study, you may contact the Institutional Review Board by calling (312) 503-9338.

**Your valuable input is crucial to evaluation of the CDRH panel process. We greatly appreciate your assistance!**

© 2024 Alam M et al. *JAMA Network Open.*

## SECTION 1: Medical Device Advisory Experience.

1. **On which Medical Device Advisory Panels have you served? (Check all that apply)**

- ☐ 1 Anesthesia and Respiratory Therapy
- ☐ 2 Circulatory System
- ☐ 3 Clinical Chemistry and Clinical Toxicology
- ☐ 4 Dental Products
- ☐ 5 Ear, Nose, and Throat
- ☐ 6 Gastroenterology-Urology
- ☐ 7 General and Plastic Surgery
- ☐ 8 General Hospital and Personal Use
- ☐ 9 Hematology and Pathology
- ☐ 10 Immunology
- ☐ 11 Medical Devices Dispute Resolution
- ☐ 12 Microbiology
- ☐ 13 Molecular and Clinical Genetics
- ☐ 14 Neurological
- ☐ 15 Obstetrics and Gynecology
- ☐ 16 Ophthalmic
- ☐ 17 Orthopaedic and Rehabilitation
- ☐ 18 Radiological

2. **How many total *terms* (often a term is 3 years) have you served on a Medical Device Advisory Panel. If you are in the middle of a term, please count this as one.**

☐

3. **How many Panel meetings did you attend *in total* as a Voting Member or Temporary Voting Member?**

☐

4. **How many *years* in total have you served on a Medical Device Advisory Panel (i.e., CDRH Panel) (please include the entire duration, even if there were no Panel meetings in a given year)**

☐

© 2024 Alam M et al. *JAMA Network Open.*

**SECTION 2: Relative Influence of Information Available for Panel Decisions**

**5. Please indicate how important each of the following sources of information is in your decision, as a member of the Panel, to recommend or not recommend approval regarding new devices:**

| | Not at all influential | Somewhat influential | Moderately influential | Very influential | Extremely influential |
|---|---|---|---|---|---|
| A. Written information read prior to the meeting. | ① | ② | ③ | ④ | ⑤ |
| B. Prior professional knowledge. | ① | ② | ③ | ④ | ⑤ |
| C. Live presentations to Panel. | ① | ② | ③ | ④ | ⑤ |
| D. Outside responses (FDA) <u>*to panel questions*</u> during the presentation. | ① | ② | ③ | ④ | ⑤ |
| E. Outside responses (industry) <u>*to Panel questions*</u> during the presentation. | ① | ② | ③ | ④ | ⑤ |
| F. Other Panel member opinions expressed <u>*during the Panel review*</u>. | ① | ② | ③ | ④ | ⑤ |
| G. Public comments made by citizens, patients and professional societies and others during the comment period. | ① | ② | ③ | ④ | ⑤ |
| H. Comparison data for existing approved devices. | ① | ② | ③ | ④ | ⑤ |

**6. In your opinion, how important are <u>*Sponsor/Company presentations*</u> to …**

| | <u>*Not*</u> important | A little important | Moderately important | Very Important |
|---|---|---|---|---|
| A. Your understanding of the risks and benefits of the device under consideration? | ① | ② | ③ | ④ |



© 2024 Alam M et al. *JAMA Network Open.*

| | | | | |
|---|---|---|---|---|
| B. Your arriving at a decision regarding approval or non-approval of the device under consideration? | ① | ② | ③ | ④ |

**7. For you, which *written information* is *most* important? (Check one)**

① Sponsor/company written or electronic information read prior to the meeting.

② FDA written or electronic information read prior to the meeting.

③ Other, please describe ____________________

**8. For you, which type of such *pre-existing relevant knowledge (e.g., prior professional knowledge)* is *most* important? (Check one)**

① Reputation of the Sponsor/Company prior professional knowledge.

② Device-under-review prior professional knowledge.

③ Other, please describe ____________________

**9. For you, which type of *live presentation to the Panel* is *most* important? (Check one)**

① Live Sponsor/Company presentation at the Panel meeting.

② Live FDA presentation at the Panel meeting.

③ Other, please describe ____________________

**10. For you, which type of *outside response to Panel questions* is *most* important? (Check one)**

① Sponsor/Company responses to questions from Panel members.

② FDA responses to questions from Panel members.

③ Other, please describe ____________________

**11. For you, which type of *other Panel member opinion expressed during the Panel review* is *most* important?**
**(Check one numbered circle – if you select 5, check as many boxes as apply)**

① Panel chair responses to FDA questions.

② General Panel chair views voiced during the discussion.

③ Panel member response to FDA questions.

④ General views of other Panel members voiced during the discussion.

⑤ Views of particular types of Panel member – please note which ones below:

- ☐1 Biostatistician(s)
- ☐2 Patient representative
- ☐3 Industry representative



© 2024 Alam M et al. *JAMA Network Open.*

☐4 Scientists/specialists (e.g., chemist or engineer expert on the device components)

☐5 Clinicians *in your own medical specialty*.

☐6 Clinicians *in medical specialties other than yours*.

⑥ Other, please describe ______________________________________________

**12. For you, which type of *public comment* from the comment period is *most* important? (Check one)**

① Organizational or group spokesperson's comments.

② Individual person's comments.

③ Other, please describe ______________________________________________

**13. For you, data from which type of *existing approved devices* is *most* important? (Check one)**

① Devices that treat similar *conditions*.

② Devices that *function* in similar ways.

③ Other, please describe ______________________________________________

**14. How do you weight *safety* versus *effectiveness* in determining suitability for approval?**

| *Effectiveness* is *much more* important ① | *Effectiveness* is *somewhat more* important ② | Both are equally important ③ | *Safety* is *somewhat more* important ④ | *Safety* is *much more* important ⑤ |
|---|---|---|---|---|

Comments:

**15. Suppose device A and device B have *equivalent* effectiveness and safety profiles. Device A is *already approved* for another indication and device B is *not*. Would you be …**

| Just as likely to approve **A or B** ① | A little more likely to approve **A** ② | Moderately more likely to approve **A** ③ | Much more likely to approve **A** ④ |
|---|---|---|---|

**16. Suppose device A and device B have *equivalent* effectiveness and safety profiles. Device A is *already approved* by regulatory authorities in an *industrialized country other than the US* (e.g., Canada, Europe, CE mark) and device B is *not*. Would you be …**

| Just as likely to approve **A or B** ① | A little more likely to approve **A** ② | Moderately more likely to approve **A** ③ | Much more likely to approve **A** ④ |
|---|---|---|---|



© 2024 Alam M et al. *JAMA Network Open.*

17. **Suppose device A serves the *__same medical purpose__* and has the __same__ effectiveness and safety profile of device B that has *__already been approved__*. Would this make you …**

| Less likely to approve **A** | Just as likely to approve **A** as if the similar device B had NOT been approved | A little more likely to approve **A** | Moderately more likely to approve **A** | Much more likely to approve **A** |
|---|---|---|---|---|
| ① | ② | ③ | ④ | ⑤ |

**SECTION 3: Pivotal Trial Research Designs**

18. **Suppose the experimental design of a pivotal trial is substandard, but the results are statistically significant. How likely are you to recommend approval based on statistical significance alone?**

| Not more likely at all | Slightly more likely | Moderately likely | Very likely |
|---|---|---|---|
| ① | ② | ③ | ④ |

19. **Assume two devices, A and B, are shown equally safe and effective in their pivotal trials. However, device *__B's pivotal trial__* had *__significant design flaws__*. How do you think *__trial design flaws__* will affect the chances of device B's approval relative to device A?**

| *No* effect of trial design flaws – only outcome significance matters | B's approval chances reduced a little relative to A | B's approval chances reduced moderately relative to A | B's approval chances reduced substantially relative to A |
|---|---|---|---|
| ① | ② | ③ | ④ |

20. **Across all the devices that you have reviewed and voted on at Panel meetings, what is the likelihood that the pivotal trials were well-designed?**

| Rarely or never (0-20%) | Infrequently (21-40%) | Sometimes (41-60%) | Often (61-80%) | Always/ almost always (81-100%) |
|---|---|---|---|---|
| ① | ② | ③ | ④ | ⑤ |

⑥No expertise in trial design – can't evaluate

21. **How helpful or counterproductive do you think it would be for the FDA to solicit the advice of Panel members regarding the appropriate design of pivotal trials?**

| Very *counter*-productive | Moderately *counter*-productive | A little *counter*-productive | Have *no* effect | A little helpful | Moderately helpful | Very helpful |
|---|---|---|---|---|---|---|
| ① | ② | ③ | ④ | ⑤ | ⑥ | ⑦ |

Comments:



© 2024 Alam M et al. *JAMA Network Open.*

**22. How helpful or counterproductive do you think it would be for the FDA to ask the Panel to review the package insert proposed by the Sponsor/Company, or to offer recommendations regarding the information that should be included in the insert?**

| Very *counter*-productive | Moderately *counter*-productive | A little *counter*-productive | Have *no* effect | A little helpful | Moderately helpful | Very helpful |
|---|---|---|---|---|---|---|
| ① | ② | ③ | ④ | ⑤ | ⑥ | ⑦ |

Comments:

**SECTION 4: Depth and Detail of Evidence Provided**

**23. Consider the types of evidence relevant to your decision to approve or dis-approve a medical device. Rate the adequacy of the typical level of detail provided for each type.**

| | Much too superficial | A little too superficial | About right | A little too detailed | Much too detailed | Too varied to say |
|---|---|---|---|---|---|---|
| A. In vitro / animal studies. | ① | ② | ③ | ④ | ⑤ | ⑥ |
| B. Research design / bio-statistical analysis of pivotal trial. | ① | ② | ③ | ④ | ⑤ | ⑥ |
| C. Epidemiologic and clinical information about condition device treats. | ① | ② | ③ | ④ | ⑤ | ⑥ |
| D. Long-term use considerations or outcomes. | ① | ② | ③ | ④ | ⑤ | ⑥ |

**24. Consider the types of evidence relevant to your decision to approve or dis-approve a medical device. Rate the adequacy of the typical level of detail provided for each type.**

| | Much too superficial | A little too superficial | About right | A little too detailed | Much too detailed | Too varied to say |
|---|---|---|---|---|---|---|
| A. Serious adverse events / patients who did unusually poorly. | ① | ② | ③ | ④ | ⑤ | ⑥ |
| B. Patients who did unusually well. | ① | ② | ③ | ④ | ⑤ | ⑥ |



© 2024 Alam M et al. *JAMA Network Open.*

| | | | | | | |
|---|---|---|---|---|---|---|
| C. *Safety* data vis-a-vis approved devices for condition. | ① | ② | ③ | ④ | ⑤ | ⑥ |
| D. *Effectiveness* data vis-a-vis approved devices for condition. | ① | ② | ③ | ④ | ⑤ | ⑥ |

| | |
|---|---|
| **25. Please note any other information here that you routinely wish you had *more of* in order to make a good decision.** | |
| **26. Please note any other information here that you routinely wish you had *less of* as you work to make a good decision.** | |

**27. Consider all the information you receive on a Panel:**

- **Pivotal trial results**
- **Results of other trials in the US and foreign countries**
- **Scientific reports and case studies**

**How often has the sum total of the scientific evidence presented to the Panel been sufficient to make you feel *very comfortable* making a decision regarding device approval or nonapproval?**

| Rarely or never (0-20%) | Infrequently (21-40%) | Sometimes (41-60%) | Often (61-80%) | Always/ almost always (81-100%) |
|---|---|---|---|---|
| ① | ② | ③ | ④ | ⑤ |

Comments:

## SECTION 5: Time Allocation

**28 Do you think the *average* time available for the following *presentations* is …**

| | Much too short | Somewhat too short | About right | Somewhat too long | Much too long |
|---|---|---|---|---|---|
| A. FDA presentation. | ① | ② | ③ | ④ | ⑤ |
| B. Sponsor/ company presentation. | ① | ② | ③ | ④ | ⑤ |
| C. Public commentary & testimony. | ① | ② | ③ | ④ | ⑤ |



© 2024 Alam M et al. *JAMA Network Open.*

**29 Do you think the *average* time available for the following *questions and discussions* is …**

| | Much too short | Somewhat too short | About right | Somewhat too long | Much too long |
|---|---|---|---|---|---|
| A. Panel time to question the FDA. | ① | ② | ③ | ④ | ⑤ |
| B. Panel time to question the sponsor/company. | ① | ② | ③ | ④ | ⑤ |
| C. Panel members discuss among themselves. | ① | ② | ③ | ④ | ⑤ |

**30. Do you think the total duration of the average Panel meeting from start to finish is …**

| Much too short | Somewhat too short | About right | Somewhat too long | Much too long |
|---|---|---|---|---|
| ① | ② | ③ | ④ | ⑤ |

**SECTION 6: Panel Composition and Process.**

**31. Do you think the *number* of *participants* comprising the average voting Panel (voting members and temporary voting members present) should be …**

| *De*creased a lot | *De*creased a little | Left as is – size about right | Increased a little | Increased a lot |
|---|---|---|---|---|
| ① | ② | ③ | ④ | ⑤ |

**32. How helpful or unhelpful do you believe it would be to have an "executive session" prior to voting in which the room was cleared except for Panel members to provide time for Panel-only discussion?**

| Very *un*helpful | Moderately *un*helpful | A little *un*helpful | Have *no* effect | A little helpful | Moderately helpful | Very helpful |
|---|---|---|---|---|---|---|
| ① | ② | ③ | ④ | ⑤ | ⑥ | ⑦ |

⑧Other, please explain: ________________________________________________

**33. If you find the above idea of an "executive session" helpful *or* unhelpful, please check all reasons why.**

**Reasons you expect it would be helpful.**

- ☐1 Allow for more *honesty* of Panel opinions.
- ☐2 Allow for more *clarity* of Panel opinions.
- ☐3 Inform better Panelist question-asking.
- ☐4 Help you decide how best to vote.

**Reasons you expect it would be *un*helpful.**

- ☐6 Add pressure to agree with majority.
- ☐7 Open opportunities to get lost in detail.
- ☐8 Prolong an already lengthy process.
- ☐9 Further cloud your voting decision.



© 2024 Alam M et al. *JAMA Network Open.*

☐5 Other, describe ____________________ | ☐10 Other, describe ____________________

**34. Suppose the decision to approve or disapprove a device is *<u>extremely divided</u>* among Panel members prior to the final vote. If *<u>you were initially among those tending toward approval</u>*, would the division …**

| Strongly *reduce* your likelihood of approving | Somewhat *reduce* your likelihood of approving | No effect | Somewhat *increase* your likelihood of approving | Strongly *increase* your likelihood of approving |
|---|---|---|---|---|
| ① | ② | ③ | ④ | ⑤ |

**35. Suppose the decision to approve or disapprove a device is *<u>extremely divided</u>* among Panel members prior to the final vote. If *<u>you were initially among those tending toward disapproval</u>*, would the division …**

| Strongly *reduce* your likelihood of approving | Somewhat *reduce* your likelihood of approving | No effect | Somewhat *increase* your likelihood of approving | Strongly *increase* your likelihood of approving |
|---|---|---|---|---|
| ① | ② | ③ | ④ | ⑤ |

**36. What is the percentage of approval votes among Panel members that *<u>you think</u>* would be appropriate for the Panel to recommend device approval?**

| Simple majority | 2/3 majority | 3/4 majority | Unanimous |
|---|---|---|---|
| ① | ② | ③ | ④ |

**37. In your opinion, what is the percentage of approval votes among Panel members *<u>the FDA</u>* thinks would be appropriate for the Panel to recommend device approval?**

| Simple majority | 2/3 majority | 3/4 majority | Unanimous |
|---|---|---|---|
| ① | ② | ③ | ④ |

**38. In your opinion, what is the percentage of approval votes among Panel members *<u>the Sponsor</u>* thinks would be appropriate for the Panel to recommend device approval?**

| Simple majority | 2/3 majority | 3/4 majority | Unanimous |
|---|---|---|---|
| ① | ② | ③ | ④ |

## SECTION 7: Federal Drug Administration (FDA)

**39. Those who approve and disapprove devices must weigh potential benefits against potential harms. *<u>Compared to senior FDA policymakers</u>* are *<u>you</u>* …**

| Much more likely to focus on potential *harms* | Moderately more likely to focus on potential *harms* | Slightly more likely to focus on potential *harms* | No tendency in either direction | Slightly more likely to focus on potential *benefits* | Moderately more likely to focus on potential *benefits* | Much more likely to focus on potential *benefits* |
|---|---|---|---|---|---|---|
| ① | ② | ③ | ④ | ⑤ | ⑥ | ⑦ |



© 2024 Alam M et al. *JAMA Network Open.*

**40. In your opinion, do FDA presentations tend to favor approval, disapproval or neither?**

| Marked tendency toward *dis*approval | Slight tendency toward *dis*approval | *No* tendency in any direction | Slight tendency toward approval | Marked tendency toward approval |
|---|---|---|---|---|
| ① | ② | ③ | ④ | ⑤ |

## SECTION 8: Demographics

**Your answers here help us analyze the data.**
**Please recall that all answers are confidential and will *NOT* be linked back to you.**

**41. Do you identify as …**

① Male

② Female

**42. Do you identify as Hispanic or Latino?**

① Yes

② No

**43. Please check all race/ethnic categories that apply to you.**

☐1 White or Caucasian

☐2 Asian

☐3 Black or African-American

☐4 Native Hawaiian or Other Pacific Islander

☐5 American Indian/ Alaska Native

☐6 Other, describe ______________________________________________

**44. In which part of the country do you primarily practice medicine?**

① Midwest – IA, IL, IN, KS, MI, MN, MO, ND, NE, OH, SD WI

② Northeast- CT, DC, DE, MA, MD, ME, NH, NJ, NY, PA, RI, VT

③ Southeast- AL, AR, FL, GA, KY, LA, MS, NC, SC, TN, VA, WV

④ Southwest – AZ, NM, OK, TX

⑤ West – AK, CA, CO, HI, ID MT, NV, OR, UT, WA, WY

⑥ Not currently in practice

**45. In what type of medical practice do you work?**

① Solo

② Single-specialty group



© 2024 Alam M et al. *JAMA Network Open.*

③ Multiple-specialty group

④ Not currently in practice

**46. Is your practice …**

① Private

② Academic

③ Government, including military or VA

④ Not currently in practice

**47. How many years has it been since you completed residency training?**

☐ Or check here ☐ if *not* applicable

**48. What proportion of your *total work time* do you allocate to patient care (vs. other work)?**

☐ % Patient Care Or check here ☐ if *not* applicable

**49. What proportion of your *patient care time* is spent with outpatients and inpatients? These proportions should sum to 100%.**

☐ % Outpatient care

☐ % Inpatient care Or check here ☐ if *not* applicable

**If there are any additional thoughts you would like to share regarding the Panel process, please provide these in the space below.**



© 2024 Alam M et al. *JAMA Network Open.*

**Thank you for completing this survey.**

**Improvement to the FDA process depends on the generous cooperation of panel members such as you. Once summary results are available, we will email them to all panel members.**



© 2024 Alam M et al. *JAMA Network Open.*

| eTable 1. Characteristics of Respondents and Nonrespondents[a] | | | |
|---|---|---|---|
| | No. (%) | | |
| | Respondents | Nonrespondent | P |
| **Gender** | | | |
| Female | 26 (40.6) | 10 (35.7) | 0.66 |
| Male | 38 (59.4) | 18 (64.3) | |
| **Region** | | | |
| Midwest | 13 (20.3) | 6 (21.4) | 0.55 |
| Northeast | 10 (15.6) | 7 (25.0) | |
| South | 30 (46.9) | 9 (32.1) | |
| West | 11 (17.2) | 6 (21.4) | |
| **Panel Served[b]** | | | |
| Anesthesia and Respiratory Therapy | 8 (12.5) | 0 | 0.10 |
| Circulatory System | 5 (7.8) | 2 (7.1) | 0.91 |
| Clinical Chemistry and Clinical Toxicology | 3 (4.7) | 2 (7.1) | 0.63 |
| Dental Products | 4 (6.3) | 2 (7.1) | 0.87 |
| Ear, Nose and Throat Panel | 5 (7.8) | 2 (7.1) | |
| Gastroenterology and Urology Panel | 7 (10.9) | 3 (10.7) | 0.98 |
| General and Plastic Surgery Panel | 7 (10.9) | 2 (7.1) | 0.57 |
| General Hospital and Personal Use Panel | 1 (1.6) | 2 (7.1) | 0.17 |
| Hematology and Pathology Devices Panel | 6 (9.4) | 1 (3.6) | 0.33 |
| Immunology Devices Panel | 1 (1.6) | 3 (10.7) | 0.08 |
| Medical Devices Dispute Resolution Panel | 5 (7.8) | 0 | 0.32 |
| Microbiology Devices Panel | 3 (4.7) | 0 | 0.55 |
| Molecular and Clinical Genetics Panel | 2 (3.1) | 1 (3.6) | 0.99 |
| Neurological Devices Panel | 2 (3.1) | 3 (10.7) | 0.16 |
| Obstetric and Gynecology Devices | 6 (9.4) | 2 (7.1) | 0.73 |
| Ophthalmic Devices Panel | 3 (4.7) | 1 (3.6) | 0.81 |
| Orthopedic and Rehabilitation Devices Panel | 4 (6.3) | 2 (7.1) | 0.87 |
| Radiological Devices Panel | 7 (10.9) | 0 | 0.10 |
| **Type of Medical Training** | | | |
| Physician | 44 (68.8) | 20 (71.4) | 0.80 |
| Non-physician | 20 (31.3) | 8 (28.6) | |
| **Race/Ethnicity[b]** (n=63) | | | |
| American Indian/ Alaska Native | 2 (3.2) | NA | |
| Asian | 10 (15.9) | NA | |
| Black or African- American | 3 (4.8) | NA | |
| Native Hawaiian or Other Pacific Islander | 1 (1.6) | NA | |
| White or Caucasian | 46 (73.0) | NA | |
| **Primary Practice Affiliation** (n=60) | | | |
| Academic | 36 (60.0) | NA | |
| Private | 11 (18.3) | NA | |
| Not currently in practice | 9 (15.0) | NA | |
| Government, including military or VA | 4 (6.7) | NA | |



© 2024 Alam M et al. *JAMA Network Open.*

| | | |
|---|---|---|
| **Hispanic or Latino** (n=63) | | |
| Yes | 4 (6.3) | NA |
| No | 59 (93.7) | NA |
| **Type of Medical Practice** (n=57) | | |
| Multiple-specialty group | 28 (49.1) | NA |
| Single-specialty group | 15 (26.3) | NA |
| Not currently in practice | 11 (19.3) | NA |
| Solo | 3 (5.3) | |
| **Total terms[c] served** | | |
| mean ± standard deviation (min-max), y | 2.2±1.2 (1-8) | NA |
| **No. of panel meetings attended[d]** | | |
| mean ± standard deviation (min-max) | 3.9±4.1 (1-19) | NA |
| **Total years served on a Medical Device Advisory** | | |
| mean ± standard deviation (min-max), y | 6.8±4.4 (1-22) | NA |
| **Years Since Completion of Residency Training** | | |
| mean ± standard deviation (min-max), y | 27.3±9.3 (3-50) | NA |
| **Proportion of total work time allocated to patient** | | |
| mean ± standard deviation (min-max), % | 61.1±25.2 (0- | NA |
| **Proportion of patient care time spent with:** | | |
| mean ± standard deviation (min-max), n=41 | | |
| Outpatient care, % | 56.8±31.2 (0- | NA |
| Inpatient care, % | 40.7±31.7 (0- | NA |

[a] The total number of respondents does not include 7 who provided incomplete responses.
[b] Respondents were asked to select all that apply.
[c] A term is often 3 years
[d] Number of Panel meetings attended as a voting or temporary voting member
[e] Compared to other work
NA denotes not available.



© 2024 Alam M et al. *JAMA Network Open.*

**eTable 2. Responses Regarding the Importance of Sponsor or Company Presentations**

| Importance of sponsor/company presentations to… | Not important | A little important | Moderately important | Very important |
|---|---|---|---|---|
| Your understanding of the risks and benefits of the device (n = 60) | 1 (1.7) | 12 (20.0) | 20 (33.3) | 27 (45.0) |
| Your arriving at a decision regarding approval or non-approval of the device (n = 60) | 2 (3.3) | 16 (26.7) | 25 (41.7) | 17 (28.3) |



© 2024 Alam M et al. *JAMA Network Open.*

**eTable 3. Respondents in Each Gender and Experience Subgroup Who Selected "Very influential" or "Extremely Influential" for the Importance of Each Source of Information in Recommending Approval or Disapproval of New Devices**

| | No. of Meetings Attended | | | Gender | | |
|---|---|---|---|---|---|---|
| **Information Source** | 1-3 | >3 | P-value[a] | Female | Male | P-value[a] |
| Written information read prior to the meeting | 27 (81.8) | 20 (83.3) | 1.00 | 20 (87.0) | 28 (80.0) | 0.72 |
| Prior professional knowledge | 24 (72.7) | 15 (62.5) | 0.41 | 17 (73.9) | 23 (65.7) | 0.51 |
| Live presentations to panel | 19 (59.4) | 21 (91.3) | 0.01 | 16 (72.7) | 25 (73.5) | 0.95 |
| Outside responses (FDA) to panel questions during the presentation | 18 (54.5) | 16 (66.7) | 0.36 | 18 (78.3) | 17 (48.6) | 0.02 |
| Outside responses (industry) to panel questions during the presentation | 11 (33.3) | 15 (62.5) | 0.03 | 10 (43.5) | 17 (48.6) | 0.70 |
| Other panel member opinions expressed during the panel review | 24 (72.7) | 18 (75.0) | 0.85 | 18 (78.3) | 25 (71.4) | 0.56 |
| Public comments made by citizens/patients/professional societies during the comment period | 5 (15.2) | 4 (16.7) | 1.00 | 3 (13.0) | 5 (14.3) | 0.89 |
| Comparison data for existing approved devices | 18 (58.1) | 15 (62.5) | 0.74 | 11 (52.4) | 23 (65.7) | 0.32 |

[a] P values from two-tailed chi-squared tests



© 2024 Alam M et al. *JAMA Network Open.*

| **eTable 4.** Most Important Sources of Information Available for Panel Decisions[a] | | |
|---|---|---|
| **Most Important Written Information (n=58)[b]** | No. (%) | P-value |
| FDA written or electronic information read prior to the meeting | 46 (79.3) | <0.001 |
| Sponsor/company written or electronic information read prior to the meeting | 13 (22.4) | |
| **Most Important Type of Pre-existing Relevant Knowledge (n=53)[c]** | | |
| Device-under-review prior professional knowledge | 51 (96.2) | <0.001 |
| Reputation of the Sponsor/Company prior professional knowledge | 2 (3.8) | |
| **Most Important Type of Live Presentation to the Panel (n=59)[d]** | | |
| Live FDA presentation at the panel meeting | 38 (64.4) | 0.02 |
| Live Sponsor/Company presentation at the panel meeting | 21 (35.6) | |
| **Most Important Type of Outside Response to Panel Questions (n=60)[b]** | | |
| Sponsor/Company responses to questions from panel members | 33 (55.0) | 0.52 |
| FDA responses to questions from panel members | 28 (46.7) | |
| **Most Important Type of Other Panel Member Opinion Expressed During the Panel Review[e,f] (n=60)** | | |
| Views of particular types of panel member: | | |
| Clinicians *in your own medical specialty*. | 24 (40.0) | |
| Scientists/Specialists (e.g., chemist or engineer expert on the device components) | 23 (38.3) | |
| Clinicians *in medical specialties other than yours.* | 22 (36.7) | |
| Biostatistician | 18 (30.0) | |
| Patient representative | 10 (16.7) | |
| Industry representative | 9 (15.0) | |
| General views of other panel members voiced during the discussion | 20 (33.3) | |
| Panel member response to FDA questions | 6 (10.0) | |
| General panel chair views voiced during the discussion. | 5 (8.3) | |
| Panel chair responses to FDA questions | 2 (3.3) | |
| **Most Important Type of Public Comment (n= 55)** | | |
| Organizational or group spokesperson's comments | 43 (78.2) | <0.001 |
| Individual person's comments | 12 (21.8) | |
| **Most Important Type of Data Regarding Existing Approved Devices (n=61)[b]** | | |
| Devices that *function* in similar ways | 33 (54.1) | 0.72 |
| Devices that treat similar *conditions* | 31 (50.8) | |

[a] Patients were instructed to “check one” answer choice for each question except where noted otherwise.
[b] Total is >100% because at least one respondent selected more than one answer choice
[c] Five respondents selected “Other” and commented: "My experience and expertise;" "Neither. As a stats mentor I have no clinical experience with devices so I'm pretty open minded;" "Published data;" "Statistical expertise;" "The quality, efficacy, safety, durability of the device and the impartial exam of the data"
[d] 1 respondent selected “Other” and commented: “Independent, critical expert presenting or reviewing the data"
[e] Respondents were asked to check one answer choice but if she/he selected “views of particular types of Panel member,” she/he was asked to select all that apply. Three respondents selected “Other” and commented: "As a statistician I often rely on Panel clinicians to help me put some of the stats concerns into clinical context;" "The



© 2024 Alam M et al. *JAMA Network Open.*

importance of types of panel members will depend on the type of questions and issues at hand, so it varies;" "Society presentation like ACOG."

[f] Five respondents selected “Other” and commented: "Patients who had treatment from the product;" "Not important;" "Physicians with expertise in the area…and in the indications for use of the subject device;" "They have little influence. Where it matters is helping me understand benefit vs risk tradeoff from a patient's perspective;" "Comments which reflect a thorough understanding of the subject matter," "I consider this segment as non-contributory to my decision"



© 2024 Alam M et al. *JAMA Network Open.*

**eTable 5. Relative Weight of Safety and Effectiveness in Determining Suitability for Approval (n=59)**

| No. (%) | | | | |
|---|---|---|---|---|
| *Effectiveness* is *much more* important | *Effectiveness* is *somewhat more* important | *Both* are *equally* important | *Safety* is *somewhat more* important | *Safety* is *much more* important |
| 0 | 6 (10.2) | 34 (57.6) | 8 (13.6) | 11 (18.6) |



© 2024 Alam M et al. *JAMA Network Open.*

**eTable 6. Responses to Questions Regarding Suitability for Approval Given the Following Hypothetical Scenarios Related to Device Safety and Efficacy (n=62)**

Scenario A: *Suppose device A and device B have equivalent effectiveness and safety profiles…*

| | No. (%) | | | |
|---|---|---|---|---|
| | Just as likely to approve **A or B** | A little more likely to approve **A** | Moderately more likely to approve **A** | Much more likely to approve **A** |
| Device A is already approved for another indication and device B is not. Would you be… | 31 (50.0) | 12 (19.4) | 10 (16.1) | 9 (14.5) |
| Device A is already approved by regulatory authorities in an industrialized country other than the US (e.g., Canada, Europe) and device B is not. Would you be… | 23 (37.1) | 17 (27.4) | 13 (21.0) | 9 (14.5) |

Scenario B: *Suppose device A serves the same medical purpose and has the same effectiveness and safety profile of device B that has already been approved.*

| | No. (%) | | | | |
|---|---|---|---|---|---|
| | Less likely to approve **A** | Just as likely to approve **A** as if the similar device B had NOT been approved | A little more likely to approve **A** | Moderately more likely to approve **A** | Much more likely to approve **A** |
| Would this make you… | 3 (4.8) | 16 (25.8) | 16 (25.8) | 12 (19.4) | 15 (24.2) |

© 2024 Alam M et al. *JAMA Network Open.*

**eTable 7. Respondents' Beliefs About Likelihood that Pivotal Trials Were Well-Designed with Regard to All Devices They Had Reviewed and Voted on at Panel Meetings (n=61), No. (%)**

| Rarely or never (0-20%) | Infrequently (21-40%) | Sometimes (41-60%) | Often (61-80%) | Always/almost always (81-100%) | No expertise in trial design- can't evaluate |
|---|---|---|---|---|---|
| 1 (1.6) | 10 (16.4) | 15 (24.6) | 22 (36.1) | 6 (9.8) | 7 (11.5) |



© 2024 Alam M et al. *JAMA Network Open.*

**eTable 8. Respondents' Attitudes Toward the Helpfulness of Including Panel Members in Specific Approval Preparation Steps**

| | No. (%) | | | | | | |
|---|---|---|---|---|---|---|---|
| | Very *counter*-productive | Moderately *counter*-productive | A little *counter*-productive | Have *no* effect | A little helpful | Moderately helpful | Very helpful |
| FDA soliciting the advice of panel members regarding the appropriate design of pivotal trials (n=62) | 0 | 0 | 5 (8.1) | 2 (3.2) | 9 (14.5) | 29 (46.8) | 17 (27.4) |
| Panel members reviewing of package insert proposed by the Sponsor/Company or to offering recommendations regarding the information that should be included in the insert (n= 64) | 0 | 2 (3.1) | 5 (7.8) | 3 (4.7) | 9 (14.1) | 28 (43.7) | 17 (26.6) |

**eTable 9. Responses to Questions Regarding Suitability for Approval Given the Following Hypothetical Scenarios Related to Pivotal Trials (n=63)**

Scenario A: *Suppose the experimental design of a pivotal trial is substandard, but the results are statistically significant.*

| | No. (%) | | | |
|---|---|---|---|---|
| | Not likely at all | Slightly likely | Moderately likely | Very likely |
| How likely are you to recommend approval based on statistical significance alone? | 39 (61.9) | 17 (27.0) | 7 (11.1) | 0 |

Scenario B: *Assume two devices, A and B, are shown equally safe and effective in their pivotal trials. However, device B's pivotal trial had significant design flaws.*

| | No. (%) | | | |
|---|---|---|---|---|
| | No effect of trial design flaws- only outcome significance matters | B's approval chances reduced a little relative to A | B's approval chances reduced moderately relative to A | B's approval chances reduced substantially relative to A |
| How do you think trial design flaws will affect the chances of device B's approval relative to device A? | 1 (1.6) | 4 (6.3) | 25 (39.7) | 33 (52.4) |

eTable 10. Respondents' Beliefs About How Often the Scientific Evidence Makes Them Feel Comfortable About Deciding Device Approval or Nonapproval

| **Question** | **Respondents, No. (%)** | | | | |
|---|---|---|---|---|---|
| | **Rarely or never (0-20%)** | **Infrequently (21%-40%)** | **Sometimes (41%-60%)** | **Often (61%-80%)** | **Always or almost always (81%-100%)** |
| How often is the sum total of the scientific evidence presented to the panel sufficient to make you feel very comfortable making a decision regarding device approval or non-approval? (n = 57) | 0 | 1 (1.7) | 13 (22.8) | 29 (50.9) | 14 (24.6) |

**eTable 11. Respondents' Beliefs about the Adequacy of the Time Allotted to Specific Segments of the Panel Meetings (n=58)**

| | No. (%) | | | | |
|---|---|---|---|---|---|
| | Much too short | Somewhat too short | About right | Somewhat too long | Much too long |
| Public commentary & testimony | 0 | 2 (3.4) | 37 (63.8) | 13 (22.4) | 6 (10.4) |
| FDA presentations | 0 | 4 (6.9) | 49 (84.5) | 5 (8.6) | 0 |
| Sponsor/ company presentation | 0 | 3 (5.2) | 50 (86.2) | 5 (8.6) | 0 |
| Panel time to question the FDA | 0 | 12 (20.7) | 46 (79.3) | 0 | 0 |
| Panel time to question the Sponsor/Company | 1 (1.7) | 12 (20.7) | 44 (75.9) | 1 (1.7) | 0 |
| Panel members discuss among themselves | 3 (5.2) | 11 (19.0) | 43 (74.1) | 1 (1.7) | 0 |
| **Total duration of the average panel meeting from start to finish** | 0 | 10 (17.2) | 44 (75.9) | 4 (6.9) | 0 |

**eFigure 1. Respondents' Beliefs About the Percentage of Approval Votes Among Panel Members that the FDA and the Sponsor/Company Think Would Be Appropriate for the Panel to Recommend Approval**

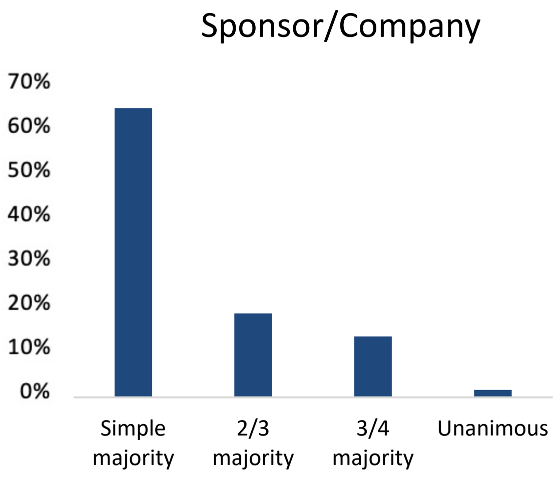


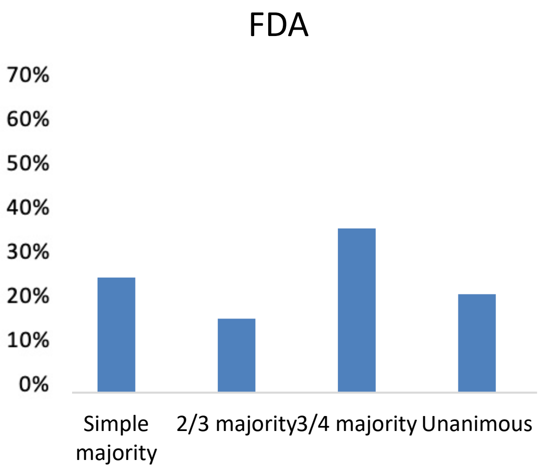

**eTable 12. Respondents' Beliefs about the Adequacy of the Number of Participants Comprising the Average Voting Panel (n=57)**

Question: Do you think the number of participants comprising the average voting panel should be…

| *De*creased a lot | *De*creased a little | Left as is (size is about right) | Increased a little | Increased a lot |
|---|---|---|---|---|
| 0 | 7 (12.3) | 45 (78.9) | 5 (8.8) | 0 |

**eTable 13. Responses to Questions Regarding Suitability for Approval Given the Following Hypothetical Scenario Related to Panel Voting (n=61)**

Scenario*: Suppose the decision to approve or disapprove a device is* ***extremely divided*** *among panel members prior to the final vote.*

| | No. (%) | | | | |
|---|---|---|---|---|---|
| | Strongly *reduce* your likelihood of approving | Somewhat *reduce* your likelihood of approving | No effect | Somewhat *increase* your likelihood of approving | Strongly *increase* your likelihood of approving |
| A. *If you were initially among those tending toward* ***approval****, would the division…* | 2 (3.3) | 19 (31.1) | 37 (60.7) | 3 (4.9) | 0 |
| B. *If you were initially among those tending toward* ***disapproval****, would the division…* | 1 (1.6) | 9 (14.7) | 37 (60.7) | 14 (23.0) | 0 |

**eTable 14. Respondents' Beliefs About the Extent to Which FDA Considers Harms versus Benefits, and is Inclined to Favor Device Approval**

No. (%)

**Question: Compared to senior FDA policymakers are you… (n=58)**

| Much more likely to focus on potential *harms* | Moderately more likely to focus on potential *harms* | Slightly more likely to focus on potential *harms* | No tendency in either direction | Slightly more likely to focus on potential *benefits* | Moderately more likely to focus on potential *benefits* | Much more likely to focus on potential *benefits* |
|---|---|---|---|---|---|---|
| 1 (1.6) | 3 (5.2) | 7 (12.1) | 22 (37.9) | 15 (25.9) | 7 (12.1) | 3 (5.2) |

**Question: Respondents' Beliefs About the Tendency of FDA Presentations to Favor Approval, Disapproval or Neither (n=55)**

| Marked tendency toward *dis*approval | Slight tendency toward *dis*approval | *No* tendency in any direction | Slight tendency toward approval | Marked tendency toward approval | - | - |
|---|---|---|---|---|---|---|
| 1 (1.8) | 11 (20.0) | 29 (52.7) | 11 (20.0) | 3 (5.5) | | |

## eFigure 2. Impact of FACA on Potential Executive Sessions

Despite panel members' desire for an executive session, that may not be possible under the Federal Advisory Committee Act, which requires all advisory committee meetings to be open to the public, 5a USC 10a, unless one of several exceptions apply, 5a U.S.C. 10(d). The exceptions, 5b USC (a)(C), include that a meeting can be closed if there would be the disclosure of personal information or confidential commercial and financial information, but these exceptions likely would not cover committee deliberations about health and safety studies, except in limited circumstances.
