# Supplementary material for: US FDA Advisory Panel Members’ Assessment of Premarket Approval Process and Suggestions for Improvement
Source: JAMA Netw Open. 2024 Oct 9;7(10):e2436066. doi: 10.1001/jamanetworkopen.2024.36066 (PMC11581643; doi:10.1001/jamanetworkopen.2024.36066)
Supplement: Supplement 2. — Data Sharing Statement [file jamanetwopen-e2436066-s002.pdf]

Data Sharing Statement

Alam. US FDA Advisory Panel Members' Assessment of Premarket Approval Process and Suggestions for Improvement. *JAMA Netw Open*. Published October 09, 2024. doi:10.1001/jamanetworkopen.2024.36066

Data
Data available: Yes
Data types: Deidentified participant data
How to access data: m-alam@northwestern.edu
When available: With publication

Supporting Documents
Document types: None

Additional Information
Who can access the data: anyone requesting the data
Types of analyses: for any purpose or for a specified purpose
Mechanisms of data availability: with a signed data access agreement
